# Developmental trajectory of care dependency in older stroke patients

**DOI:** 10.3389/fneur.2024.1374477

**Published:** 2024-05-20

**Authors:** Qinger Lin, Xiaohang Dong, Tianrong Huang, Hongzhen Zhou

**Affiliations:** ^1^Department of Nursing, Nanfang Hospital, Southern Medical University, Guangzhou, China; ^2^School of Nursing, Southern Medical University, Guangzhou, China; ^3^Department of Neurology, Nanfang Hospital, Southern Medical University, Guangzhou, China; ^4^Department of Neurology, The Third Affiliated Hospital of Southern Medical University, Guangzhou, China

**Keywords:** stroke, comorbidity, dependency, older adult, developmental trajectory

## Abstract

**Background:**

Stroke is the leading cause of death in China. Older stroke survivors often have other chronic conditions, not only musculoskeletal deterioration due to age, but also changes in body image that can be brought on by stroke and other diseases, making them unable to take good care of themselves and dependent on others. The degree of dependency affects the rehabilitation progress of stroke survivors and shows dynamic changes that need to be recognized.

**Objectives:**

This study investigates the trajectory of dependency changes in older stroke patients with comorbidities and analyze the influencing factors.

**Methods:**

Grounded in the Chronic Illness Trajectory Framework (CITF), a longitudinal study was conducted from February 2023 to October 2023, tracking 312 older stroke patients with comorbidities admitted to two tertiary hospitals in Guangzhou. Care dependency levels were assessed using Care Dependency Scale on admission day 5 (T0), at discharge (T1), 1 month post-discharge (T2), and 3 months post-discharge (T3). Growth Mixture Model were utilized to identify trajectory categories, and both univariate analysis and multivariate logistic regression methods were employed to explore factors associated with different developmental trajectories.

**Results:**

A total of four developmental trajectories were fitted, C1 (high independence-slow increased group, 52.0%), C2 (moderate independence-rapid increased group, 13.0%), C3 (moderate independence-slow increased group, 25.0%), and C4 (low independence-increased and decreased group, 10.0%). Length of hospital stay, place of residence, level of social support, residual functional impairments, NIHSS score, and BI index independently influence the trajectory categories.

**Conclusion:**

There is heterogeneity in care dependency among older stroke patients with comorbidities. Most patients gradually reduce their dependency and become more independent, but others remain dependent for an extended period of time. It is recommended to focus on patients who live in rural areas, have low social support, have high admission NIHSS scores and have residual functional impairment, and provide them with personalized continuity of care and rehabilitation services in order to reduce care dependency and the burden of care, and to improve patients’ quality of life.

## 1 Introduction

Stroke is the first cause of death in China (1). Ischemic stroke, accounting for approximately 86.8% of all stroke types in the population aged 40 and above, is characterized by high recurrence, disability, and mortality rates. Factors such as hypertension, diabetes, hyperlipidemia, heart disease, and atherosclerosis contribute significantly to the increased risk of ischemic stroke. These are also major chronic diseases that pose a significant threat to public health, thus making the prevention and treatment of ischemic stroke crucial (2, 3).

As of 2022, the population of individuals aged 60 and above in China exceeded 280 million, accounting for 19.8% of the total population (4). The rapid aging of the population has led to a significant increase in the population requiring complex care, highlighting the importance of promoting healthy aging and creating elderly-friendly environments to alleviate the burden of care in China. Stroke is one of the primary causes of disability in older adults, and the disease causes, for example, movement disorders, swallowing disorders, and cognitive disorders that increase the level of dependency in the lives of older adults. This not only significantly increases the burden of long-term care but also imposes a heavy socioeconomic burden (5, 6).

Chronic health conditions are common among older adults. In China, the prevalence of chronic disease comorbidity among older adults reaches 65.16%, and the majority of them suffer from two chronic diseases (7). Compared to those with only one chronic illness, individuals with two or more chronic diseases face a greater threat to their life safety and quality of life (8). A study revealed that at least 90% of middle-aged and older adults experiencing ischemic stroke suffer from at least one other chronic disease (9). Older stroke patients often concurrently experience other underlying health issues, which not only significantly impact the patients themselves but also impose economic burdens and psychological stress on their families and society (10, 11). This is because, on one hand, the trauma and functional impairments resulting from stroke severely affect the patients’ quality of life; On the other hand, the pain and fear brought about by comorbidities, as well as the prevalent sense of helplessness among older adults, lead them to seek support from their families and society, making them inclined to rely on others (12). Therefore, it is essential to understand the current status of care dependency among older stroke patients with comorbidities and to implement corresponding care measures.

In our study, the concept of care dependency is derived from the theories of human need and self-care (13, 14). Care dependency represents a specific form of reliance, which involves subjective needs for care support to compensate for deficiencies in self-care. Therefore, it is essential to assess patients’ psychological and social behaviors (15, 16). In previous longitudinal studies focusing on care dependency levels among stroke patients, the authors have noted that patients’ levels of care dependency exhibit dynamic changes (17). Thus, hospitals and communities should continuously monitor patients’ care dependency to adjust care measures in a timely manner.

Traditional analysis methods for depicting the overall developmental trajectory in research assume that all subjects follow the same trajectory, but recent studies have shown that the population does not follow the same development trajectory, and there are unobservable subgroups in the population, each of which possesses its own different growth parameters (18). Growth Mixture Model (GMM) can reflect both inter-individual differences at different time points and the continuity and trends of individual changes (19, 20). GMM has been widely applied in nursing, with research topics focusing on population quality of life, cognitive function, and psychological status. By constructing GMM, different quality of life trajectories during the 12-month recovery process of stroke survivors were identified. The results indicate that the quality of life trajectory of stroke survivors is related to the burden, anxiety, and depression of their caregivers. It is suggested that healthcare professionals pay more attention to and intervene with survivors and their caregivers exhibiting moderately low quality of life trajectories (21). By constructing GMM, the changing trajectory of cognitive function in Chinese middle-aged and elderly individuals was estimated. The results show that Chinese middle-aged and elderly individuals exhibit three heterogeneous trajectories of cognitive function, with an overall trend of gradual decline, emphasizing the need to focus on groups with lower education levels and lower self-care abilities (22). In summary, GMM can effectively address the shortcomings of traditional growth models in exploring population heterogeneity.

In this study, we will conduct a longitudinal study to construct Growth Mixture Model to identify the different developmental trajectories and influencing factors of care dependency in older ischemic stroke patients with comorbidities, which will help healthcare professionals to provide personalized care plans, better nursing guidance and the basis for developing quality continuity of care services for patients.

## 2 Materials and methods

### 2.1 Study design

This was a follow-up study. We selected ischemic stroke patients hospitalized in two tertiary care hospitals in Guangzhou from February 2023 to October 2023 by convenience sampling method, and invited those who met the inclusion criteria to participate in the study by combining medical record screening and bedside assessment of patients’ conditions during hospitalization. Detailed information about the study background, follow-up time, and assessment procedures were introduced to the participants by the researchers, and patients’ consent was obtained and signed an informed consent form.

### 2.2 Setting and sample

The inclusion criteria were as follows: ① age ≥ 60 years; ② patients with first-ever ischemic stroke with a hospital stay of ≥5 days; ③ meeting the diagnostic criteria in “Diagnostic Highlights of Various Major Cerebrovascular Diseases in China 2019” (23) and confirmed by CT or MRI examination; The primary clinical diagnosis is ischemic stroke; ④ Combine at least one chronic disease that aligns with the International Classification of Diseases (ICD-11); ⑤ stable vital signs and clear consciousness. There is some ability to communicate and understand, and the diagnosis by the physician is stable enough to participate in the study; and ⑥ voluntary participation in this study. Exclusion criteria were as follows: ① severe aphasia; ② severe cognitive impairment or mini-mental state examination (MMSE) score < 10; ③ requesting to withdraw from the study; ④ refusal or missed follow-up calls; ⑤ developing other severe illnesses or experiencing exacerbation leading to death.

According to the Bayesian Information Criterion (BIC) (24, 25), when using BIC as the primary indicator for model selection, the minimum sample size required for growth mixture models with follow-up time points of 4 or more is 200 cases.

### 2.3 Instruments

#### 2.3.1 General information questionnaire

The questionnaire was designed by the researchers themselves to collect data including patient age, gender, BMI, education level, marital status, primary caregiver, place of residence, average monthly household income, payment, stroke site and type, residual functional impairment, admission BI index (Barthel Index), length of hospital stay, and post-discharge destination.

#### 2.3.2 Care dependency scale

The Care Dependency Scale (CDS), originally proposed by Dutch nursing researcher Dijkstra, converts the 14 basic human needs proposed by nursing theorist Henderson into 15 assessment items (16). In 2014, Zhang sinicized the CDS and tested its reliability in an older population. The scale used the Likert five-point scale, with scores of 1–5 representing 5 degrees from complete dependency to complete independence in order; the total score range was 15–75, with a rating of <25 being completely dependent, 25–44 being overwhelmingly dependent, 45–59 being partially dependent, 60–69 being less dependent, and > 69 being almost independent, with lower scores indicating a greater degree of dependency. The Cronbach’s α coefficient for the Chinese version of the Care Dependency Scale was 0.959, with inter-rater reliability KaPPa values of 0.84–0.899 and retest reliability of 0.83–0.90 (26).

#### 2.3.3 National Institutes of Health Stroke Scale

It was originally developed in 1989, the NIHSS includes the following domains: level of consciousness, eye movements, integrity of visual fields, facial movements, arm and leg muscle strength, sensation, coordination, language, speech and neglect. Each impairment is scored on an ordinal scale ranging from 0 to 2, 0 to 3, or 0 to 4. Item scores are summed to a total score ranging from 0 to 42 (the higher the score, the more severe the stroke) (27). The Cronbach’s α coefficient of the Chinese version of the scale is 0.77. Our study classified stroke severity based on NIHSS scores: 0–7 points as mild stroke, 8–16 points as moderate stroke, and > 16 points as severe stroke (28).

#### 2.3.4 Cumulative illness rating scale for the geriatric

The Cumulative Rating Scale for Geriatric Disease (CIRS-G) evolved from the Cumulative Illness Rating Scale (CIRS) and was modified by Miller in 1991 to better suit the assessment of the older population, mainly by separating the hematopoietic system from the vascular system in the original scale entries and making it a separate system for assessment, with detailed scoring methods for each system (29). In order to further increase the applicability of the scale, in 1995, Parmelee adjusted the scale catalog and changed the scale to a scale of 1–5 (30). In 2008, Salvi updated and revised the guidelines for use (31). The CIRS-G currently consists of 14 systems, each assessed on a 0–4 scale of severity, each system is scored as follows: 0 (none), no impairment to that organ/system; 1 (mild), impairment does not interfere with normal activity; treatment may or may not be required; prognosis is excellent; 2 (moderate), impairment interferes with normal activity, treatment is needed, prognosis is good; 3 (severe), impairment is disabling, treatment is urgently needed, prognosis is guarded; 4 (extremely severe), impairment is life-threatening, treatment is urgent or of no avail; poor prognosis. The Cronbach’s α coefficient was 0.83, and an inter-rater reliability of 0.81 (32). Evaluation indicators are as follows:

(1) The most widely used evaluation index is the TSC (total score) (33), which is the sum of the total scores of the 14 systems, with a total score range of 0–56. (2) The severity index is the mean score of the 13 items of the above CIRS-G excluding psychiatric/behavioral [severity index = total score of the 13 items of the CIRS excluding psychiatric (behavioral)/13]. (3) Comorbidity Index refers to the number of items with a score of ≥2 out of 14 items (if six items score ≥ 2, the co-morbidity index is 6).

#### 2.3.5 Social support rating scale

Social support rating scale was developed by Xiao in 1994 to detect the extent to which individuals receive psychological support in their social life and the utilization of that support (34). The scale has 10 items, including three dimensions of objective support (three items), subjective support (four items), and utilization of social support (three items). The higher the score, the higher the level of social support. The Cronbach’s α coefficient for the scale was 0.90.

#### 2.3.6 Mini-mental state examination

The MMSE was first developed by Folstein in 1975, which mainly includes five aspects: orientation, memory, recall, attention and calculation, and language ability, with a total of 19 items and a total score of 30 points, and the higher the score, the better the cognitive function (35). The Cronbach’s α coefficient of the Chinese version of the scale is 0.833, and the retest reliability is 0.924 (36).

#### 2.3.7 Self-rating depression scale

The SDS was developed by Zung in 1965, and scale contains 20 items. Higher total scores represent more severe symptoms of depression (37). The Cronbach’s α coefficient of the Chinese version of SDS is 0.86.

### 2.4 The selection of follow-up time points

Our research theoretical framework is based on the Chronic Illness Trajectory Framework (CITF). In 1991, Corbin and Strauss proposed the concept of CITF, which not only helps nursing personnel understand the disease trajectory of chronic illnesses to maintain patients’ stable quality of life but also provides direction for nursing practice, teaching, research, and policy reform (38). Currently, the CITF has been used in the management of patients with heart disease (39, 40), cancer (41), diabetes (42), stroke (43), and other chronic diseases (44). Based on CITF, Kirkevold proposed staging of the disease trajectory for stroke patients. Kirkevold’s research indicates that the disease trajectory of stroke patients includes onset (1–7 days post-onset), early recovery (1–8 weeks post-onset), sustained recovery (8 weeks to 6 months post-onset), and semi-stable period (6 months post-onset) (45). We will adopt this trajectory staging, focusing on stroke onset, early recovery, and sustained periods, with investigation times ranging from 5 days after the patient’s onset to 3 months after discharge. The investigation will be conducted at four time points: day 5 after admission (T0), the day of discharge (T1), 1 month after discharge (T2), and 3 months after discharge (T3).

### 2.5 Data collection

**Figure 1:**
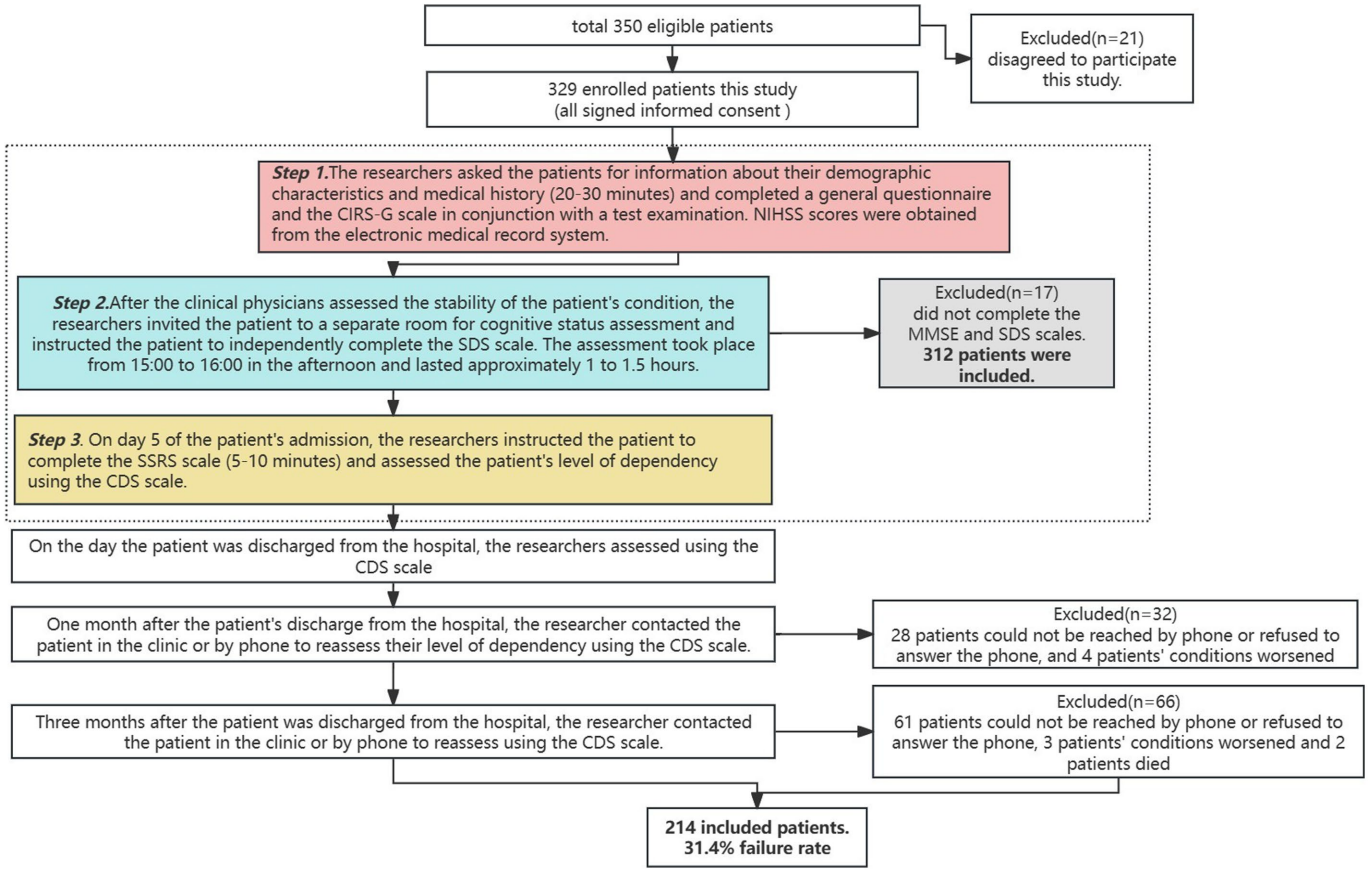
Flowchart of data collection process.

The procedures for data collection were as follows:

① After patients were admitted to the hospital, the researcher sent invitations to patients who met the inclusion criteria and all patients signed an informed consent form. Patients were assessed by the researcher and a clinician for their overall condition, as well as asked for personal information, past medical history and scored using the NIHSS. In conjunction with later laboratory testing, the researcher recorded the pertinent data and performed a detailed determination of comorbidities. ② After the clinical physicians assessed the stability of the patient’s condition, the researchers invited the patient to a separate room for cognitive status assessment and instructed the patient to independently complete the SDS scale. The assessment took place from 15:00 to 16:00 in the afternoon and lasted approximately 1–1.5 h. ③ The researcher used the CDS to score the degree of dependency of the patients on the 5th day of admission (T0), on the day of discharge (T1), 1 month after discharge (T2), and 3 months after discharge (T3) by outpatient or telephone follow-up, respectively (Figure 1).

The aforementioned researcher and medical staff have undergone unified training, possess the necessary evaluation credentials, are familiar with the employed scales, and are capable of using them skillfully. The data collection process was conducted using a unified guideline to ensure the uniformity of data collection and management.

### 2.6 Data analysis

Growth Mixture Models were fitted using Mplus 8.3, and classes 1–5 were selected sequentially for analysis.

Model fitting indicators include:

1. Akaike information criteria (AIC), Bayesian information criteria (BIC), and adjusted BIC (aBIC), the smaller the value is, the better the model fits.
2. Entropy value: the value range is 0–1, the closer to 1 means the more accurate classification.
3. Bootstrap-based likelihood ratio test (BLRT) are used for model comparison, and when the test is significant (*p* < 0.05), it means that the k^th^ model is better than the *k*-1^th^ model. The best-fitting model was selected by considering the above indicators in the fitting results of each type of model together.

IBM SPSS 25.0 was used for data description and analysis. The measurement data conforming to normal distribution were expressed as (M ± SD), and one-way ANOVA was used for comparison between multiple groups; non-normally distributed metric data are represented by the median and interquartile range M (P25, P75), and non-parametric tests are used for comparisons between multiple groups. The count data were expressed as number of cases and percentages, and the χ^2^ test or Fisher’s exact probability method was used for comparison between multiple groups. Multivariate logistic regression analysis was used to explore the factors influencing care dependency in older ischemic stroke patients with comorbidities, and when *p* < 0.05 indicates a statistically significant difference.

## 3 Results

### 3.1 Participant’s demographics

During the baseline period, 312 patients were initially included. During the follow-up process, 98 patients were lost due to phone refusal, worsening of the condition, and other reasons. Finally, a total of 214 patients who completed the full follow-up were included, with a loss to follow-up rate of 31.4% (Figure 1).

All 214 patients completed all the scales and questionnaires. 214 patients were aged 60–90, with 142 males (66.4%), 179 married (83.6%), 75 with primary school education or below (35.0%), 138 spouses as caregivers (64.5%), 157 resided in urban areas (73.4%), and 183 people were covered by medical insurance (85.5%); the length of hospital stay was (11.66 ± 3.70) days, with 139 patients discharged and returned home (65.0%). The top five comorbidities were hypertension in 175 cases (81.7%), vascular diseases in 109 cases (50.9%), endocrine diseases in 90 cases (42.1%), respiratory diseases in 85 cases (39.7%), and heart diseases in 35 cases (16.4%).

### 3.2 Four measurements of CDS

The CDS scores for the four measurements were (52.64 ± 14.71), (57.22 ± 11.83), (59.98 ± 10.65), and (61.70 ± 11.72), respectively. There is a positive correlation between the total scores of the four measurements (*r* = 0.89, 0.82, 0.68, *p* < 0.001), which meets the premise of follow-up research.

### 3.3 Total and individual item scores for care dependency at four time points

The descriptions of each item in the Chinese version of the CDS are as follows:

Item 1, Eating, refers to meeting one’s own dietary needs; Item 2, Incontinence, refers to independently managing bowel and bladder movements; Item 3, Body posture, refers to adopting appropriate body positions independently while lying down or sitting; Item 4, Mobility, refers to independently moving out of bed and walking; Item 5, Day/night pattern, refers to maintaining a normal day-night rhythm without disturbances; Item 6, Dressed and undresses, refers to independently putting on and taking off clothing; Item 7, Body temperature, refers to regulating one’s body temperature through measures such as adjusting clothing; Item 8, Hygiene, refers to completing personal hygiene tasks including washing face, brushing teeth, and combing hair; Item 9, Avoidance of danger, refers to ensuring personal safety; Item 10, Communication, refers to engaging in communication with others; Item 11, Contact with others, refers to initiating, maintaining, and terminating social interactions; Item 12, Sense of rules and values, refers to adhering to societal norms and rules independently; Item 13, Daily activities, refers to organizing daily activities independently; Item 14, Recreational activities, refers to participating in external activities independently; and Item 15, Learning ability, refers to self-directed learning and acquisition of knowledge and skills (26).

The physiological function dimension comprises items 1 through 9, while the psychosocial function dimension includes items 10 through 15. For the scores of different items at different time points, please refer to Table 1.

**Table 1:** Scores of each items at different time points.

CDS scores for each item at admission day 5 (*N* = 214)			
Item	Minimum value	Maximum value	Score (*-x*± s)
CDS scores	17	75	52.64 ± 14.71
Physiological function	10	45	32.97 ± 9.54
Eating	1	5	3.96 ± 1.38
Incontinence	1	5	4.07 ± 1.38
Body posture	1	5	3.86 ± 1.21
Mobility	1	5	2.58 ± 1.38
Day/night pattern	1	5	4.69 ± 0.78
Dressed and undresses	1	5	3.62 ± 1.39
Body temperature	1	5	3.95 ± 1.21
Hygiene	1	5	3.47 ± 1.42
Avoidance of danger	1	5	2.77 ± 1.24
Psychosocial function	6	30	20.85 ± 5.78
Communication	1	5	4.00 ± 1.08
Contact with others	1	5	3.73 ± 1.15
Sense of rules and values	1	5	4.09 ± 1.04
Daily activities	1	5	3.21 ± 1.24
Recreational activities	1	5	2.77 ± 1.17
Learning ability	1	5	3.04 ± 1.01

CDS scores for each item on the day of discharge (*N* = 214)			
Item	Minimum value	Maximum value	Score (*-x* ± *s*)
CDS score	20	75	57.22 ± 11.83
Physiological function	12	25	35.28 ± 7.38
Eating	1	5	4.17 ± 1.05
Incontinence	1	5	4.27 ± 1.03
Body posture	1	5	4.02 ± 1.00
Mobility	1	5	3.06 ± 1.15
Day/night pattern	2	5	4.72 ± 0.62
Dressed and undresses	1	5	3.91 ± 1.12
Body temperature	1	55	4.13 ± 0.99
Hygiene	1	5	3.72 ± 1.16
Avoidance of danger	1	5	3.26 ± 1.08
psychosocial function	8	30	21.74 ± 5.10
Communication	2	5	4.07 ± 0.89
Contact with others	1	5	3.86 ± 1.01
Sense of rules and values	1	5	4.10 ± 0.91
Daily activities	1	5	3.54 ± 1.11
Recreational activities	1	5	3.14 ± 1.05
Learning ability	1	5	3.02 ± 0.93

CDS scores for each item at 1 month post-discharge (*N* = 214)			
Item	Minimum value	Maximum value	Score (*-x* ± *s*)
CDS score	25	75	59.98 ± 10.65
Physiological function	16	45	37.18 ± 6.23
Eating	2	5	4.41 ± 0.82
Incontinence	2	5	4.53 ± 0.77
Body posture	2	5	4.20 ± 0.87
Mobility	1	5	3.32 ± 1.07
Day/night pattern	3	5	4.80 ± 0.50
Dressed and undresses	2	5	4.14 ± 0.91
Body temperature	2	5	4.35 ± 0.86
Hygiene	1	5	3.96 ± 1.04
Avoidance of danger	1	5	3.48 ± 1.01
Psychosocial function	7	30	22.64 ± 4.92
Communication	2	5	4.21 ± 0.81
Contact with others	1	5	4.04 ± 0.96
Sense of rules and values	1	5	4.17 ± 0.91
Daily activities	1	5	3.73 ± 1.06
Recreational activities	1	5	3.36 ± 0.97
Learning ability	1	5	3.12 ± 0.94

CDS scores for each item at 3 months post-discharge (*N* = 214)			
Item	Minimum value	Maximum value	Score (*-x* ± *s*)
CDS score	21	75	61.70 ± 11.72
Physiological function	14	45	38.08 ± 6.62
Eating	2	5	4.46 ± 0.81
Incontinence	2	5	4.57 ± 0.78
Body posture	1	5	4.32 ± 0.91
Mobility	1	5	3.57 ± 1.03
Day/night pattern	2	5	4.79 ± 0.52
Dressed and undresses	2	5	4.23 ± 0.90
Body temperature	1	5	4.40 ± 0.91
Hygiene	1	5	4.10 ± 1.02
Avoidance of danger	1	5	3.64 ± 1.04
Psychosocial function	7	30	23.43 ± 5.49
Communication	2	5	4.32 ± 0.87
Contact with others	1	5	4.21 ± 0.99
Sense of rules and values	1	5	4.25 ± 0.98
Daily activities	1	5	3.94 ± 1.06
Recreational activities	1	5	3.50 ± 1.09
Learning ability	1	5	3.21 ± 1.07

### 3.4 Developmental trajectory of care dependency

#### 3.4.1 Model fitting for care dependency score

We set up Growth Mixture Models for 1–5 classes to fit the data. The results showed that the AIC, BIC, and aBIC values of the five models decreased sequentially, with Entropy > 0.80 and BLRT < 0.001. However, one of the five classes in the model is less than 5%, and the sample may not be representative. Considering the explainability of the model, the final model is selected as the four classes (four developmental trajectories), which account for 52.0, 13.0, 25.0, and 10.0% of the total, respectively (Table 2, Model A).

**Table 2:** Model fitting results.

Model (A): fitting results of care dependency						
Model	AIC	BIC	aBIC	BLRT	Entropy	Classes probability
1	5639.67	5669.97	5641.45	*p* < 0.001	-	-
2	5586.77	5627.19	5589.16	*p* < 0.001	0.872	0.13/0.87
3	5544.89	5595.38	5547.84	*p* < 0.001	0.917	0.65/0.24/0.9
**4**	**5524.77**	**5584.77**	**5527.73**	***p* < 0.001**	**0.865**	**0.25/0.10/0.13/0.52**
5	5504.70	5575.39	5508.84	*p* < 0.001	0.902	0.16/0.10/0.02/0.22/0.50
**Model (B): fitting results of the physiological functional dimension**						
**Model**	**AIC**	**BIC**	**aBIC**	**BLRT**	**Entropy**	**Classes probability**
1	4826.92	4857.21	4828.69	-	-	-
2	4763.22	4803.62	4765.59	*p* < 0.001	0.903	0.86/0.13
3	4733.63	4784.12	4736.59	*p* < 0.001	0.828	0.21/0.66/0.13
**4**	**4714.26**	**4774.85**	**4717.81**	***p* < 0.001**	**0.841**	**0.62/0.08/0.13/0.17**
5	4705.56	4776.25	4709.70	*p* = 0.19	0.867	0.02/0.18/0.19/0.11/0.60
**Model (C): fitting results of the psychosocial functional dimension**						
**Model**	**AIC**	**BIC**	**aBIC**	**BLRT**	**Entropy**	**Classes probability**
1	4309.09	4339.38	4310.86	-	-	-
2	4278.80	4319.19	4281.17	*p* < 0.001	0.816	0.80/0.20
**3**	**4268.36**	**4318.85**	**4271.31**	***p* = 0.03**	**0.828**	**0.25/0.19/0.66**
4	4256.08	4316.66	4259.63	*p* = 0.01	0.855	0.03/0.09/0.24/0.64
5	4238.84	4309.52	4242.98	*p* < 0.001	0.833	0.09/0.02/0.15/0.26/0.48

AIC, Akaike information criteria; BIC, Bayesian information criteria; aBIC, adjusted BIC; BLRT, Bootstrap-based likelihood ratio test. The bold values indicate that we have selected the model of that class among the model classes.

**Figure 2:**
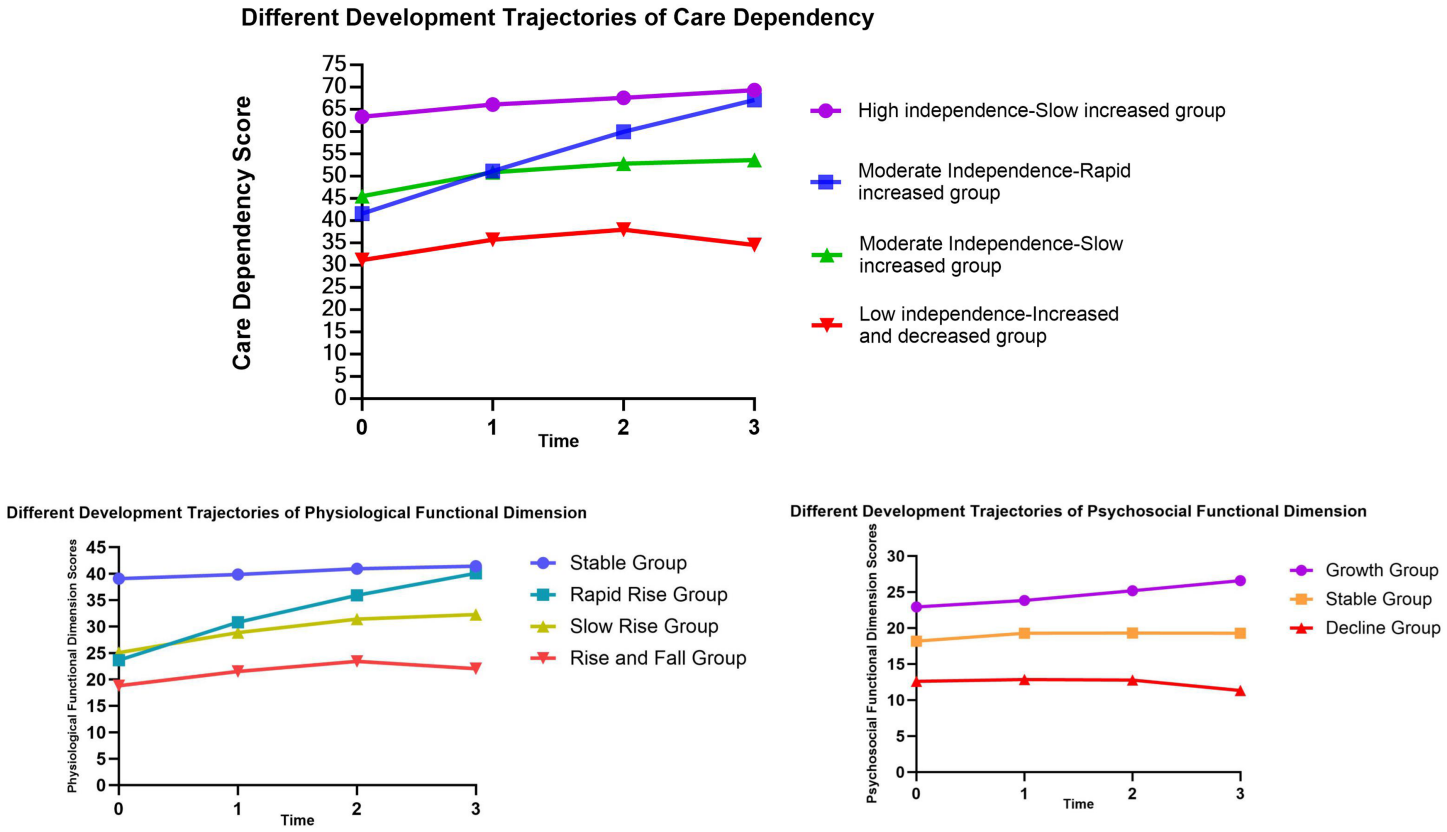
Different development trajectories of care dependency and Its two dimensions.

The GMM for the four categories indicates that in C1, the intercept value (α) is the highest, with a slope (β) value greater than 1 (*p* < 0.001), suggesting the highest level of independence in C1, named the “High independence-Slow increased group”; in C2, the intercept value is moderate, with the highest slope value (*p* < 0.001), indicating moderate independence in C2, with scores rapidly increasing over time, named the “Moderate Independence-Rapid increased group”; in C3, the intercept value is moderate, with a slope value greater than 1 (*p* < 0.001), indicating initially moderate independence in C3, with scores continuing to rise over time, but with a lower slope compared to C2, hence named the “Moderate independence-Slow increased group”; in C4, the intercept value is the smallest, with a slope value less than 1 (*p* = 0.66), indicating slow rise in scores, and a declining trend in the T2-T3 phase, suggesting the highest dependency and lowest independence in C4, named the “Low independence-Increased and decreased group” as shown in Table 3, Model A. Based on these four categories, a developmental trajectory of care dependency total scores is formed, as depicted in Figure 2.

**Table 3:** Model parameter estimation results.

The parameter estimation results of Model (A)						
Classes		Intercept			Slope	
	Estimated value	Standard error	*p*	Estimated value	Standard error	*p*
C1	64.24	1.12	<0.001	1.66	0.36	<0.001
C2	42.88	2.33	<0.001	8.24	0.71	<0.001
C3	48.44	1.44	<0.001	1.93	0.47	<0.001
C4	35.38	1.93	<0.001	0.23	0.53	0.660
**The parameter estimation results of Model (B)**						
**Classes**	**Intercept**			**Slope**		
	**Estimated value**	**Standard error**	***p***	**Estimated value**	**Standard error**	***p***
C1	39.14	0.75	<0.001	0.82	0.23	<0.001
C2	25.15	2.06	<0.001	5.20	0.81	<0.001
C3	26.42	1.41	<0.001	2.23	0.52	<0.001
C4	20.55	1.38	<0.001	0.97	0.31	0.002
**The parameter estimation results of Model (C)**						
**Classes**	**Intercept**			**Slope**		
	**Estimated value**	**Standard error**	***p***	**Estimated value**	**Standard error**	***p***
C1	22.70	0.48	<0.001	1.27	0.12	<0.001
C2	18.73	0.57	<0.001	0.24	0.24	0.308
C3	13.28	1.52	<0.001	−0.51	0.31	0.101

#### 3.4.2 Model fitting of physiological function dimensions

Growth mixture models with 1–5 classes were set up to fit free estimation models to the data on physiological function scores. The results showed that the AIC, BIC, and aBIC values of the five-class models decreased sequentially, and the Entropy values were all greater than 0.8, which indicated higher classification accuracy. The BLRT values of the first four categories are less than 0.001. The BLRT value of the five-class model is greater than 0.05, indicating that the five-category model is not better than the four-category model. Taking all indicators into consideration, we select the 4-class model as the best model, with the four classes comprising 62, 8, 13, and 17% of the total population, respectively. The fitted data for each class are detailed in Table 2, Model B.

The model for the four latent classes shows that in C1, the intercept value (α) is the highest, with a slope value (β) less than 1 (*p* < 0.001), indicating the highest scores in C1, exhibiting an overall stable trend, named as the “Stable Group”; in C2, the α value is moderate, with the highest β value (*p* < 0.001), indicating a rapid increase in physiological function scores over time, named the “Rapid Rise Group”; in C3, the α value is moderate, with β value greater than 1 but small (*p* < 0.001), indicating a steady increase in scores over time, named the “Slow Rise Group”; in C4, the α value is the smallest, with β value less than 1 (*p* = 0.002), showing an overall stable trend, while the developmental trajectory exhibits an initial increase followed by a decrease, named the “Rise and Fall Group,” as detailed in the Table 3, Model B. Based on these four categories, a developmental trajectory of physiological function scores is formed, as shown in the Figure 2.

#### 3.4.3 Model fitting of psychosocial functioning dimensions

Growth mixture models with 1–5 classes were set up to fit free estimation models to the data on psychosocial functioning scores. The results showed that the AIC, BIC, and aBIC values of the five-class model decreased sequentially, the Entropy values were all greater than 0.8, indicating higher classification accuracy, and the BLRT values were all less than 0.05, as the probability of 1 of the 4 classes was less than 5%, and the sample was not representative. Combining the above indicators and practical considerations, the model with three classes was selected as the best model, and the three classes accounted for 25, 19, and 66% of the total, respectively. The fitted data are detailed in the Table 2, Model C.

The model of the three latent categories shows that the intercept value(α) of group 1 is the largest, and the slope (β) value is greater than 1 (*p* < 0.001), indicating that C1 has the highest score, and the overall trend is upward, named as the “Growth Group”; In C2, the α value is medium and the β value is less than 1 (*p* = 0.308), indicating that the overall trend of C2’s score is stable, named as Stable Group; Conversely, C3 displays the lowest intercept value, with β value less than 0 (*p* = 0.101), indicating an overall descending trend in scores, named as the “Decline Group,” as detailed in the Table 3, Model C. The developmental trajectory of psychosocial function scores based on these three groups is depicted in Figure 2.

### 3.5 Influencing factors between different developmental trajectories

#### 3.5.1 One-way ANOVA

The age, BMI, NIHSS score, length of hospital stay, type of functional impairment, caregiver, place of residence, BI Index, comorbidity score, comorbidity index, severity index, cognitive state (MMSE), depressive state (SDS), social support (SSRS), and post discharge destination of older stroke patients with different developmental trajectories were compared, and the differences were statistically significant (*p* < 0.05). Among the comorbidities, hypertension and endocrine system diseases showed statistically significant differences in the four developmental trajectories (*p* < 0.05) (Table 4).

**Table 4:** Results of a univariate analysis of the developmental trajectory of care dependency in older stroke patients with comorbidities (*N* = 214).

Variables	Total	C1 (*n* = 112)	C2 (*n* = 28)	C3 (*n* = 54)	C4 (*n* = 20)	*F*/χ^2^/*H*	^*^ *p*
Age	65.0 (60.0,71.0)	65.21 (60,68.75)	65.29 (60.0,69.0)	67.50 (61.0,74.0)	65.50 (60.25,72.75)	9.96^c^	0.019
M (P25,P75)							
Sex							
Man (%)	142 (66.4)	75 (67.0)	19 (67.9)	34 (63.0)	14 (70.0)	0.46^b^	0.942
Woman (%)	72 (33.6)	37 (33.0)	9 (32.1)	20 (37.0)	6 (30.0)		
BMI (kg/m^2^, M ± SD)	23.82 ± 3.13	24.26 ± 3.20	24.13 ± 2.83	23.23 ± 3.13	22.51 ± 2.75	2.69^a^	0.047
Educational level							
Primary education and below (%)	75 (35.0)	36 (32.1)	10 (35.7)	17 (31.5)	12 (60.0)		
Junior high school (%)	91 (42.5)	49 (43.8)	13 (46.4)	24 (44.4)	5 (25.0)	17.47^b^	0.031
High school (%)	29 (13.6)	20 (17.9)	0	6 (11.1)	3 (15.0)		
College degree or Above (%)	19 (8.9)	7 (6.3)	5 (17.9)	7 (13.0)	0		
Marital status							
Married (%)	179 (83.6)	99 (88.4)	22 (78.6)	42 (77.8)	16 (80.0)		
Widowed (%)	34 (15.9)	13 (11.6)	5 (17.9)	12 (22.2)	4 (20.0)	8.87^b^	0.147
Divorced (%)	1 (0.5)	0	1 (3.6)	0	0		
Residence							
Urban areas (%)	157 (73.4)	82 (73.2)	24 (85.7)	42 (77.8)	9 (45.0)	10.96^b^	0.011
Rural areas (%)	57 (26.6)	30 (26.8)	4 (14.3)	12 (22.2)	11 (55.0)		
Caregiver							
Spouse (%)	138 (64.5)	83 (74.1)	17 (60.7)	30 (55.6)	8 (40.0)		
Adult child (%)	67 (31.3)	24 (21.4)	10 (35.7)	22 (40.7)	11 (55.0)	13.09^b^	0.041
Other (%)	9 (4.2)	5 (4.5)	1 (3.6)	2 (3.7)	1 (5.0)		
Payment method							
Self-financed (%)	31 (14.5)	18 (16.1)	3 (10.7)	5 (9.3)	5 (25.0)	3.41^b^	0.334
Medical insurance payments (%)	183 (85.5)	94 (83.9)	25 (89.3)	49 (90.7)	15 (75.0)		
BI Index							
100(%)	25 (11.7)	23 (21.5)	0	2 (3.7)	0		
46–99(%)	119 (55.6)	79 (70.5)	13 (46.4)	23 (42.6)	4 (20.0)	71.22^b^	<0.001
0–45(%)	70 (32.7)	10 (9.0)	15 (53.6)	29 (53.7)	16 (80.0)		
Stroke site							
Left hemisphere (%)	81 (37.9)	50 (44.6)	11 (39.3)	14 (25.9)	6 (30.0)		
Right hemisphere (%)	81 (37.9)	34 (30.4)	11 (39.3)	26 (48.1)	10 (50.0)		
Cerebellar (%)	7 (3.3)	5 (4.5)	0	2 (3.7)	0	15.76^b^	0.203
Brainstem (%)	27 (12.6)	10 (8.9)	4 (14.3)	10 (18.5)	3 (15.0)		
Other site (%)	18 (8.4)	13 (11.6)	2 (7.1)	2 (3.7)	1 (5.0)		
Stroke type							
Large artery atherosclerosis (%)	85 (39.7)	29 (25.9)	16 (57.1)	29 (53.7)	11 (55.0)		
Small artery occlusion (%)	120 (56.1)	83 (74.1)	11 (39.3)	21 (38.9)	5 (25.0)	41.35^b^	<0.001
Other (%)	9 (4.2)	0	1 (3.6)	4 (7.4)	4 (20.0)		
Type of functional impairment							
None (%)	69 (32.2)	49 (43.8)	8 (28.6)	9 (16.7)	3 (15.0)		
One (%)	122 (57.1)	57 (50.9)	18 (64.3)	38 (70.4)	9 (45.0)	27.69^b^	<0.001
Two or more (%)	23 (10.7)	6 (5.3)	2 (7.1)	7 (12.9)	8 (40.0)		
NIHSS score							
≤7 (%)	191 (89.3)	111 (99.1)	27 (96.4)	41 (75.9)	12 (60.0)	37.96^b^	<0.001
8–16 (%)	23 (10.7)	1 (0.9)	1 (3.6)	13 (24.1)	8 (40.0)		
CIRS-G (M ± SD)	9 (7,11)	8 (7,10)	10.50 (7,15)	9.50 (8,11)	12 (8.25,16)	19.67^c^	0.012
Comorbidity index M (P25, P75)	3.39 (2.75,4)	3 (2,4)	3.50 (2.25,5)	3 (3,4)	4 (3.25,5)	14.78^c^	0.002
Severity index M (P25, P75)	0.7 (0.54,0.85)	0.62 (0.54,0.77)	0.81 (0.54,1.08)	0.69 (0.54,0.85)	0.85 (0.63,1.15)	15.93^c^	<0.001
MMSE (M ± SD)	19.91 ± 5.55	21.29 ± 5.24	19.88 ± 4.93	18.86 ± 5.30	15.03 ± 5.69	9.04^a^	<0.001
SDS (M ± SD)	43.82 ± 11.68	41.97 ± 11.79	42.52 ± 11.95	46.41 ± 10.35	49.00 ± 12.09	3.35^a^	0.020
SSRS (M ± SD)	38.86 ± 2.76	39.13 ± 2.50	40.46 ± 2.80	38.09 ± 2.64	37.20 ± 3.05	8.06^a^	<0.001
Length of hospitalization M (P25, P75)	11.0 (8.0,14.0)	10.24 (7.0,14.0)	13.46 (10.25,15.0)	14 (10.0,15.0)	14.25 (11.75,15.0)	34.20^c^	<0.001
Post-discharge destination							
Home (%)	139 (65.0)	85 (75.9)	17 (60.7)	29 (53.7)	11 (55.0)		
Rehabilitation facilities (%)	75 (35.0)	27 (24.1)	11 (39.3)	25 (46.3)	9 (45.0)	9.99^b^	0.018

C1: high independence-slow increased group, C2: moderate independence-rapid increase group, C3: moderate independence-slow increased group, C4: low independence-increased and decreased group; ^a^One-way ANOVA; ^b^Chi-square; ^c^K-S test. CIRS-G, Cumulative Illness Rating Scale for the Geriatric; NIHSS, National Institutes of Health Stroke Scale; MMSE, Mini-mental state examination; SDS, Self-Rating Depression Scale; SSRS, Social Support Rating Scale; M, Mean; SD, Standard deviation.

#### 3.5.2 Multivariate logistic regression analysis

The statistically significant variables identified from the univariate analysis were taken as independent variables, with the four developmental trajectories of care dependency as the dependent variable. The High independence-slow increased group (C1) served as the reference group for conducting multivariate logistic regression analysis. The categorical variables are assigned as follows: for education level, 1 = primary school and below, 2 = middle school, 3 = high school, 4 = college and above; for caregivers, 1 = spouse, 2 = children, 3 = other relatives; for place of residence, 1 = urban, 2 = rural; for admission NIHSS score, 1 = 0–7 points, 2 = 8–16 points; for admission BI index, 1 = 100, 2 = 46–99, 3 = 0–45; for stroke type, 1 = large artery atherosclerosis, 2 = small artery occlusion, 3 = other; for functional impairment, 1 = no, 2 = 1 type, 2 = 2 types or more; for post-discharge destination, 1 = home, 2 = rehabilitation institution. In multiple categorical variables, the variables with a value of 1 are used as reference variables.

The results of the regression analysis showed that the log-likelihood ratio χ^2^ was 195.497, *p* < 0.001. Length of hospital stay, place of residence, SSRS score (social support), functional impairment, NIHSS score, and BI index were independent influencing factors for different trajectories (*p* < 0.05) (Table 5).

**Table 5:** Results of multivariate logistic regression analysis of factors influencing the developmental trajectory of care dependency in older stroke patients with comorbidities (*N* = 214).

Dependent variable	Independent variable	Categories	β	S.E	Wald χ^2^	^*^ *p*	OR (95%CI)
C2	SSRS	-	0.26	0.11	5.76	0.049	1.254 (1.00–1.573)
	Length of hospitalization	-	0.24	0.08	9.05	0.022	1.203 (1.026–1.410)
	Comorbidity index	-	−0.90	0.44	4.26	0.039	0.407 (0.174–0.956)
C3	NIHSS score	<7	−2.53	1.21	4.39	0.036	0.080 (0.007–0.849)
	SSRS	-	−0.19	0.09	4.83	0.028	0.830 (0.702–0.980)
	BI	0–45	−3.11	1.04	8.97	0.003	0.045 (0.006–0.340)
C4	Functional impairment	One	3.58	1.58	5.09	0.024	35.146 (1.596–73.876)
	SSRS	-	−0.66	0.21	9.68	0.002	0.519 (0.343–0.784)
	Residence	Rural area	−4.91	1.59	9.58	0.002	0.007 (0.001–0.165)

With C1 as the control group; C1: high independence-slow increased group, C2: moderate independence-rapid increase group, C3: moderate independence-slow increased group, and C4: low independence-increased and decreased group. NIHSS, National Institutes of Health Stroke Scale; SSRS, Social Support Rating Scale; BI, Barthel Index; β, Regression coefficient; S.E, Standard error; Waldχ^2^ = (β/S.E)^2^; OR, Odds ratio.

Specifically, compared to the High independence-slow increased group (C1), the influencing factors for older ischemic stroke patients with comorbidities developing into the Moderate independence-rapid increased group (C2) were social support level, length of hospital stay, and comorbidity index. The probability of being classified into the Moderate independence-rapid increased group (C2) is 1.254 times higher (OR = 1.254, 95% CI: 1.00, 1.573) for those with higher social support level, 1.203 times higher (OR = 1.203, 95% CI: 1.026, 1.410) for those with longer hospital stays, and 0.407 times lower (OR = 0.407, 95% CI: 0.174, 0.956) for those with a higher comorbidity index compared to the high independence-slow increased group (C1), indicating that higher social support level, longer hospital stays, and lower comorbidity index were more likely to be classified into the Moderate independence-rapid increased group (C2).

Compared to the High independence-slow increased group (C1), the influencing factors for older ischemic stroke patients with comorbidities to develop into the Moderate independence-slow increased group (C3) include the level of social support, NIHSS score, and admission BI index. The probabilities of being classified into the Moderate independence-slow increased group (C3) for those with higher social support, NIHSS scores of 0–7, and BI index of 0–45 are 0.83 times (OR = 0.830, 95%CI: 0.702, 0.980), 0.08 times (OR = 0.080, 95%CI: 0.007, 0.849), and 0.045 times (OR = 0.045, 95%CI: 0.006, 0.340) respectively, compared to the High independence-slow increased group (C1). This indicates that lower social support, higher NIHSS scores, and BI index make it more likely for individuals to be classified into the Moderate independence-slow increased group (C3).

Compared to the High independence-slow increased group (C1), the influencing factors for older ischemic stroke patients with comorbidities developing into the Low independence-increased and decreased group (C4) were social support level, place of residence, functional impairment, and NIHSS score. The probability of being classified into the Low independence-increased and decreased group (C4) for those with higher social support, living in urban areas, having a type of residual functional disability, and an NIHSS score of 0–7 is 0.519 times (OR = 0.519, 95%CI: 0.343, 0.784), 0.007 times (OR = 0.007, 95%CI: 0.001, 0.165), 35.146 times (OR = 35.146, 95%CI: 1.596, 73.876), and 0.005 times (OR = 0.005, 95%CI: 0.001, 0.182), respectively, compared to the High independence-slow increased group (C1). This indicates that those with lower social support, residing in rural areas, having one type of functional impairment, and NIHSS score greater than 7 are more likely to be classified into the Low independence-increased and decreased group (C4).

## 4 Discussion

### 4.1 The overall developmental trend of care dependency

During hospitalization, the total score for care dependency was (52.64 ± 14.71), indicating a moderate level of dependency. Items with lower scores included mobility, recreational activities, and avoidance of danger. Due to comorbid chronic conditions such as high blood pressure, heart disease, or diabetes, older stroke patients with poorly controlled conditions may experience dizziness and weakness in their arms and legs, so they tend to rely on others for greater fear of falling or bedridden. Some patients may reduce their activities and outings to lower the incidence of adverse events (46). This highlights the importance of not only monitoring patients’ physiological functions but also addressing their safety risks.

At discharge, the total score for care dependency was (57.22 ± 11.83), indicating a moderate level of dependency. Items with lower scores included learning ability, mobility, and recreational activities. This suggests that older ischemic stroke patients, following treatment and care during hospitalization, experienced improved or stabilized conditions, with some degree of neurological function recovery and enhanced self-care abilities. However, due to age-related decline in physical function and the impact of the disease, older stroke patients may experience decreased responsiveness, cognitive abilities, and memory (47). Meanwhile, some patients still present varying degrees and types of functional impairments at discharge, making them unable to maintain normal learning and daily activities, and they still need further nursing guidance and support after discharge.

After discharge, the care dependency score at 1 and 3 months were (59.98 ± 10.65) and (61.70 ± 11.72), respectively, indicating a lower level of dependency. Lower scores were observed in items such as learning ability, recreational activities, and mobility. The overall care dependency scores showed significant improvement compared to the early stages of the disease but remained lower than previous study (48). This difference may be attributed to the inclusion of patients with comorbidities in our study. The presence of comorbidities exacerbates both the physiological and psychological trauma experienced by older stroke patients, resulting in higher levels of dependency among this population, necessitating increased attention and care. The majority of patients entering stroke rehabilitation have been able to complete basic self-care activities and meet basic physiological and safety needs, while high-level needs such as social interaction, recreational activities, and learning knowledge have not been adequately met (49).

### 4.2 The care dependency of patients exhibits different developmental trajectories

Our study found population heterogeneity in care dependency among older stroke patients with comorbidities. We constructed the Growth Mixture Model to fit four trajectories of heterogeneity of care dependency from the 5th day of admission to 3 months after discharge, namely, High independence-Slow increased group (C1), Moderate independence-Rapid increase group (C2), Moderate independence-Slow increased group (C3), and Low independence-Increased and decreased group (C4), which could more accurately reflect the changes in the trajectory of their care dependence.

52.0% of the patients were in the High independence-Slow increased group, accounting for the largest proportion, and the care dependency score gradually increased during the follow-up period, with the dependency gradually decreased to complete independence, indicating that most patients were able to self-adjust to the disease within 3 months after discharge, which was consistent with the results of previous studies (50). In addition, 25.0% of patients were in the Moderate independence-Slow increased group. Compared with patients in the high-independence group, this group of patients had more severe stroke, lower BI index, higher NIHSS score and comorbidities score, and moderate dependency at the base period. However, during the follow-up period, they also showed changes in the degree of dependency improvement, indicating that these patients also had good self-adjustment ability in the face of disease recovery. In addition, 13.0% of patients were in the Moderate independence-Rapid increase group, suggesting that although patients in this group were moderately independent during baseline, dependency improved rapidly over time and approached full independence at 3 months after discharge, similar to the findings in Han (51). There were 10.0% of patients in the Low independence-Increased and decreased group, who had low independence at baseline, then increased independence slightly at 1 month after discharge, and decreased independence at 3 months after discharge, suggesting that these patients need to focus on their health and needs after discharge and provide continuity of care plan at discharge.

We conducted an analysis of the scores depicting the changes in patient care dependency across two dimensions, and the results revealed:

1 The scores in the physiological function dimension are divided into four subgroups: Stable group, Rapid rise group, Slow rise group, and Rise and fall group. The subgroup classification of scores in the physiological function dimension is consistent with the overall scores of the scale. Physiological function dimension scores were divided into four subgroups: stable group, rapid increase group, stable increase group, and increase–decrease group. The subgroup classification of physiological function dimension scores was consistent with overall scores.

Patients in the Stable group had the highest and stable physiological function scores, and stroke caused the least physiological function impact in this group of patients. Patients in the Rapid rise group and Slow Rise group had lower baseline physiological function scores. During the T1–T3 period, the Rapid rise group showed a significant increase in scores, indicating a notable reduction in physiological function dependency, while the Slow rise group exhibited only a slight increase. This suggests that some older patients tend to rely on others due to factors such as physical frailty and residual functional impairments. Patients in the Rise and fall group showed a slight increase in physiological function scores from T1 to T2, followed by a decline at T3, indicating a minor enhancement in independence shortly after discharge, followed by a return to initial levels. This may be attributed to patients returning to familiar living environments post-discharge, normalizing routines, and experiencing a relatively rapid recovery period with greater hopes for rehabilitation and overall positive attitudes. But, after 1 month post-discharge, the rate of functional recovery in various aspects declined, leading to psychological burdens and concerns about disease progression or recurrent stroke, resulting in fluctuations in psychological states. This group of patients represents a priority for continued care.

2 The scores in the psychological-social function dimension can be divided into three subgroups: Growth group, Stable group, and Decline group. Patients in the Growth and Stable groups generally experience some degree of improvement in dependency levels as treatment, care, and rehabilitation progress. Patients in the Decline group show a consistent decline in psychological-social function from the onset of illness to the recovery period. This is consistent with previous research (52), suggesting that psychological impairments persist post-discharge and may not recover as rapidly as physical functions, particularly within the first three months after discharge. During this time, patients often experience significant fluctuations in their psychological state, which greatly impact their quality of life. In addition, stroke-induced cognitive impairment is a possible cause; cognitive impairment is a common sequela after stroke and can persist even after seemingly successful neurological recovery, which in turn affects the performance of daily living abilities.

### 4.3 Factors influencing the developmental trajectory of care dependency in older stroke patient with comorbidities

#### 4.3.1 Patients with longer hospital stays, higher levels of social support, and lower comorbidity index are more likely to be classified into moderate independence-rapid increased group

In our study, the level of social support satisfaction among older stroke patients with comorbidities was moderately above average, mirroring previous research findings (53). A well-established social support system can offer improved social security, welfare, and convenient access to medical and healthcare services. Moreover, older adults can derive essential social companionship, instrumental aid, and emotional solace from their social networks to mitigate negative emotions and enhance their capacity to cope with illness more effectively (54, 55). In addition, this may correlate with patients undergoing early and sustained rehabilitation training during their hospitalization. Early rehabilitation plays a pivotal role in mitigating the burden of stroke-related disabilities. Guidelines emphasize that ischemic stroke patients with stable conditions who undergo neurorehabilitation training earlier and for extended durations experience better recovery of physical function and heightened independence (56, 57).

The presence of multiple chronic conditions is strongly associated with increased dependency, particularly notable in cases of neurological disorders. Compared to individuals without comorbidities, those with concurrent conditions experience a greater decline in their quality of life index as the number of comorbidities rises (58). Among stroke patients, cardiovascular metabolic diseases emerge as the primary comorbidity pattern. In our study, over 80% of patients had hypertension, over 50% had vascular diseases (mainly dyslipidemia), over 40% had endocrine disorders (mostly diabetes), and 16% had cardiac issues (predominantly atrial fibrillation). “Hypertension + diabetes” currently represents the most prevalent comorbidity combination in ischemic stroke cases, exacerbating patient weakness through disruptions in metabolism and cardiovascular function (9, 59). Dyslipidemia is a recognized risk factor for stroke, with each unit increase in blood cholesterol concentration significantly raising the risk of ischemic stroke. Atrial fibrillation is a well-established predictor of poor post-stroke outcomes, markedly increasing the risk and disease burden, leading to more frequent severe strokes and more pronounced neurological damage compared to those without atrial fibrillation (60). Studies have shown that cardiovascular metabolic diseases increase the likelihood of cognitive impairment in elderly patients, with elderly atrial fibrillation patients maintaining a persistently high dependency rate 3 years post-stroke (61, 62). In summary, comorbidities represent more than just the sum of two diseases; they present greater diagnostic challenges and poorer treatment outcomes for patients. Healthcare providers should tailor post-stroke interventions based on comorbidity profiles to reduce additional mortality rates.

#### 4.3.2 Patients with lower levels of social support, higher NIHSS scores, and BI indexes were more likely to be classified in the moderate independence-slow increased group

The preceding discussion emphasized the significance of social support for older patients. In addition, our study found that patients with higher NIHSS scores on admission were more likely to enter the group with lower independence. Baseline NIHSS scores are critical for predicting ischemic stroke prognosis. The duration and extent of brain tissue injury in patients with acute ischemic stroke are closely related to poor prognosis. The longer the duration and more severe the degree of brain tissue injury, the worse the speed of recovery and prognosis of patients (63, 64).

BI index reflects a patient’s daily life capabilities. Dependency on activities of daily living is a common aftermath of stroke, persisting in 35% of stroke survivors (65). Even patients with mild stroke experience functional dependence in daily life and persistent unmet rehabilitation needs (66). Therefore, in addition to the severity of stroke, it is also necessary to assess daily functioning in the early post-stroke period to estimate prognosis. For most patients, recovery in daily life abilities typically occurs within the initial 6 weeks post-stroke, correlating with the stroke’s severity at onset. Nevertheless, in the later stages of stroke recovery, improvements in daily life abilities are generally limited. Studies have shown that patients experiencing dependency in activities of daily living in the first week post-stroke continue to exhibit this dependency at 6 months and 3 years post-stroke (67, 68). Recent studies have also reported that early ability to perform activities of daily living is a good predictor of long-term outcome in stroke patients (69). This suggests that attention should be paid to patients’ early ability to perform activities of daily living and to keep in touch with their rehabilitation needs and the formulation of care measures.

#### 4.3.3 Patients with lower levels of social support, residing in rural areas, and residual functional impairment are more likely to be classified into the low independence-increased and decreased group

Older adults in rural areas are an important population group for stroke, with higher rates of morbidity, disability, and mortality than older adults in urban areas (70). Studies indicate that the burden of risk factors for stroke is higher in rural areas than in urban areas, and that hypertension, diabetes and heart disease are more prevalent in rural areas than in urban areas (71). Stroke patients in rural areas may be insensitive to disease perception due to financial constraints or lack of stroke-related knowledge, leading to delay in seeking medical treatment and missing the optimal period of treatment and rehabilitation (72). The study points out that the health care resources and facilities of the environment in which older adults live are significantly associated with care dependency (73). In addition to the resource needs of the elderly, a point that cannot be ignored is that the current social changes and demographic changes are changing the family structure and the size of the elderly population, and that a portion of the rural elderly are separated from their adult children for long periods of time and lack other ways of emotional support, which may be one of the reasons influencing the low level of social support and the lower independence of the patients who reside in rural areas for a long period of time after the onset of the disease (74). This suggests that healthcare professionals, in addition to paying close attention to the physical self-care ability of this category of patients, also need to be assessed by increasing psychosocial dependence. When the patient’s condition is in stable condition, through the questionnaire assessment, combined with the patient and his/her family interviews, to understand the patient’s psychological condition, timely detection of psychological problems, so that the patients with psychosocial dependence understand their own psychological or social interaction problems, so that they consciously seek for solutions from health care professionals, and obtain psychological counseling interventions as early as possible. Previous studies have included CDS in the follow-up management program, which has enhanced the awareness of stroke patients’ active follow-up and improved the quality of life of stroke patients, which further suggests the importance of dynamic psychosocial follow-up assessment and intervention guidance for stroke patients (75). In the meantime, family and friends of the patients were encouraged to give care to the patients and organize “patient association” and other mutual exchanges to improve the patients’ psychological resilience and confidence in overcoming the disease.

The presence of multiple functional impairments after stroke is associated with a variety of factors, including older age, comorbidities (69), cognitive impairment (76), and severity at stroke onset (63). The majority (>80%) of residual functional impairments in stroke survivors are related to motor dysfunction. Elderly patients experience physiological decline and musculoskeletal degeneration with age, leading to reduced capacity to respond to external pressures (77). Most ischemic stroke survivors with residual functional impairments require long-term rehabilitation and care. However, those who do not receive early rehabilitation training or fail to adhere to rehabilitation programs may not achieve favorable outcomes. Thus, motor dysfunction remains a significant factor affecting the independence of patients for a considerable period after the onset of stroke (78). Older patients who undergo changes in body image are more prone to develop pessimistic attitudes, leading to less determination to change their motor behavior and weaker self-awareness for change (79). Some patients may even be discriminated against and rejected because their physical behavior is different from normal people, resulting in a sense of shame and a negative attitude toward treatment, which greatly hinders the recovery process of stroke patients (80). Therefore, such patients need to be closely monitored and assessed. On one hand, healthcare professionals should not only inform patients about the importance of early rehabilitation training once their condition stabilizes but also pay close attention to their emotional changes, provide appropriate comfort and encouragement, enhance their confidence in change, and improve their compliance with rehabilitation training (81). A multidisciplinary team approach can be employed to promote patients’ recovery from various aspects including functional training, psychological support, and nutrition (50, 82). On the other hand, the rehabilitation needs and support for stroke survivors after discharge mainly rely on caregivers or family members. Therefore, it is crucial to engage caregivers or family members in the care process, allowing them to participate in shared supervision (83). Healthcare professionals should provide necessary disease knowledge dissemination and rehabilitation exercise guidance, and offer them supportive assistance. Previous studies have suggested establishing continuity care teams and involving community healthcare professionals in the team to encourage patients to make full use of community resources to promote disease recovery (49). The recent implementation of regional medical consortia has effectively met the medical needs of stroke rehabilitation patients by establishing collaborative relationships between hospitals and communities (84–86).

## 5 Conclusion and limitation

From the 5th day of admission to 3 months post-discharge, the overall trajectory of care dependency in older stroke patients with comorbidities shows a downward trend. With the progression of treatment, care, and rehabilitation, the patients’ dependency decreases overall, while their independence gradually increases. Through Growth Mixture Model, it was revealed that there exists population heterogeneity in the overall care dependency among older stroke patients with comorbidities, delineating four distinct developmental trajectories. Length of hospital stay, place of residence, level of social support, residual functional impairments, NIHSS score, and BI index independently influence the trajectory categories. Healthcare professionals can identify patients belonging to different developmental groups based on characteristic differences and formulate nursing plans tailored to different stages for patients in different groups. It is recommended to focus on patients who live in rural areas, have low social support, have high admission NIHSS scores and have residual functional impairment, and provide them with personalized continuity of care and rehabilitation services in order to reduce care dependency and the burden of care, and to improve patients’ quality of life.

Our study has several limitations. Firstly, due to constraints in manpower, resources, and funding, we were only able to collect complete follow-up data from 214 cases. The small sample size may lead to reduced statistical power and limit the generalizability of our findings. To mitigate this issue, we adopted a Bayesian approach when selecting the optimal model. The Bayesian approach offers advantages in reducing the reliance on sample size and better aligning with the researchers’ theoretical framework, thus minimizing errors associated with small sample sizes. Additionally, our follow-up period extended only up to 3 months post-discharge. In future research endeavors, we are committed to addressing these limitations by striving to increase the sample size and extend the follow-up duration. This will allow for a more comprehensive understanding of our study population.

## Data availability statement

The raw data supporting the conclusions of this article will be made available by the authors, without undue reservation.

## Ethics statement

The studies involving humans were approved by Ethics Committees of the Nanfang Hospital of Southern Medical University (approval number: NFEC-2022-419). The studies were conducted in accordance with the local legislation and institutional requirements. Written informed consent for participation in this study was provided by the participants’ legal guardians/next of kin.

## Author contributions

QL: Conceptualization, Data curation, Formal analysis, Investigation, Methodology, Software, Writing – original draft, Writing – review & editing. XD: Data curation, Investigation, Writing – original draft. TH: Data curation, Investigation, Writing – review & editing, Software. HZ: Conceptualization, Formal analysis, Funding acquisition, Methodology, Software, Supervision, Validation, Writing – review & editing.
